# PSA-Stratified Performance of [^18^F]DCFPyL PET/CT in Biochemically Recurrent Prostate Cancer Patients under Androgen Deprivation Therapy

**DOI:** 10.3390/diagnostics12092212

**Published:** 2022-09-13

**Authors:** Sara Harsini, Don Wilson, François Bénard

**Affiliations:** 1 BC Cancer Research Institute, 675 West 10th Ave, Vancouver, BC V5Z 1L3, Canada; 2 Department of Radiology, University of British Columbia, Vancouver, BC V5Z 1M9, Canada

**Keywords:** prostate cancer, biochemical recurrence, prostate-specific membrane antigen, androgen deprivation therapy

## Abstract

Based on in vitro studies, it is known that androgen deprivation therapy (ADT) increases prostate-specific membrane antigen (PSMA) expression on prostate cancer (PCa) cells. However, ADT also has cytoreductive effects which can decrease lesion size. The present evaluation was conducted to further analyze the influence of ongoing ADT on [^18^F]DCFPyL positron emission tomography/computed tomography (PET/CT) performance in the setting of biochemically recurrent PCa. We retrospectively evaluated two groups of PCa patients, previously treated with radical intent, who had undergone [^18^F]DCFPyL PET/CT because of biochemical relapse with a minimum PSA level of 0.4 ng/mL. One group consisted of 95 patients under ADT at the time of the PET examination, and the other consisted of 445 patients not receiving ADT at the time of PET/CT. The uptake characteristics of the cardiac blood pool, liver, parotid glands, and five most active lesions were measured and compared between these two groups. The overall detection rate of [^18^F]DCFPyL PET/CT in patients under ADT at the time of imaging was significantly higher than patients not under ADT (91.6% vs. 80.4%, *p*-value = 0.007). However, the PSA-stratified differences in detection rates between patients with and without ADT did not reach statistical significance. Except for the maximal standardized uptake values corrected for lean body mass (SULmax) in the PSA range of 1 to <2 ng/mL, the intensity and volume of [^18^F]DCFPyL accumulation were higher in patients with ADT compared to the patients without. Statistical significance was attained for the SULmax in PSA range of 0.5 to <1 ng/mL (*p*-value = 0.0004) and metabolic tumor volume (MTV) in all PSA ranges (*p*-values of 0.0005 to 0.03). No significant difference was observed for radiotracer uptake in normal organs between the two groups with and without ADT. In this study population with biochemical recurrence of PCa and measurable PSA, ongoing ADT at the time of [^18^F]DCFPyL PET/CT imaging was associated with higher radiotracer uptake and overall lesion detection rate. This could be due in part to the more aggressive disease phenotype in patients with ongoing ADT.

## 1. Introduction

The prostate-specific membrane antigen (PSMA), a transmembrane glycoprotein overexpressed on nearly all prostate cancer (PCa) cells, has gained prominence as a molecular target for imaging and therapy of PCa [1,2,3]. The PSMA receptor exerts its role in oncogenic signaling in the PCa cells via acting on the glutamate receptors and activation of the Pi3K and Akt growth pathways [4,5,6]. Mostly suggested by in vitro studies and animal models, the administration of androgen deprivation therapy (ADT) appears to increase PSMA expression [7,8]. Although there are some investigations showing an increase in maximum standardized uptake value (SUVmax) of PSMA-positive lesions after the initiation of ADT in human subjects [7], the impact of ADT on PSMA positron emission tomography (PET) results was deemed heterogeneous in a few recent studies [9,10].

Given the paucity of data on the influence of ADT on [^18^F]DCFPyL PET intensity and performance, the present study aimed to assess if the tumor visibility and detection rates of [^18^F]DCFPyL PET/CT in different PSA subgroups are affected by ADT in patients with PCa previously treated with radical intent and who presented biochemical relapse. The goal was to determine whether it is appropriate to refer patients with ongoing ADT, who are progressing by PSA, for [^18^F]DCFPyL PET/CT. To this end, we assessed the detection rates as well as the tumor uptake characteristics in two groups of patients with and without ADT at the time of PET/CT scanning.

## 2. Materials and Methods

### 2.1. Participants

This is a retrospective analysis of data from an ongoing investigator-initiated clinical trial (clinicaltrials.gov NCT02899312). Five hundred and forty prostate cancer patients with biochemical evidence of recurrent disease after initial therapy, including radical prostatectomy and radiotherapy with or without ADT, were included in this study between May 2017 and January 2020. Biochemical recurrence was defined as a PSA greater than 0.4 ng/mL or as a PSA level of greater than 2 ng/mL above the nadir after initial curative therapy with radiation therapy. Exclusion criteria were: medical instability, inability to provide written consent, exceeding the safe weight of the PET/CT bed (204.5 kg), inability to fit through the PET/CT bore (70 cm diameter), inability to lie supine for imaging, or ECOG > 2. The study did not exclude participants undergoing ADT, either recently instituted or on ongoing or intermittent regimens. Patients enrolled in this study were divided into two groups: 95 patients who were under ADT at the time of the PET/CT and 445 patients who were not receiving any additional systemic therapy. Patients in the second group were not under ADT for at least 6 months. To exclude a possible influence of different PSA levels on the sensitivity of imaging with [^18^F]DCFPyL, patients in these two groups were arbitrarily categorized according to their PSA level: 0.4 ≤ PSA < 0.5 ng/mL, 0.5 ≤ PSA < 1 ng/mL, 1 ≤ PSA < 2 ng/mL, 2 ≤ PSA < 10 ng/mL, and 10 ≤ PSA ng/mL. All imaging studies were performed in the course of the clinical diagnostic workup. This study has been approved by the UBC/BC Cancer Research Ethics Board and by Health Canada. Written informed consent was obtained from all participants prior to enrollment in the study.

### 2.2. Radiotracer and Imaging

[^18^F]DCFPyL was synthesized in accordance with a previously published method [11]. The administered activity was scaled by body weight (range: 237–474 MBq), allowing for a 10% variation in target activity. After a 120 min uptake period, participants were imaged from top-of-head to mid-thigh on a Discovery PET/CT 600 or 690 (GE Healthcare). A CT scan for localization and attenuation correction (120 kV, automatic mA selection (30–200 mA range), and noise index of 20) was acquired. PET data were acquired instantly after the CT data over 2–4 min/bed position, adjusted for patient girth, and reconstructed using the ordered-subset expectation maximization algorithm and point-spread-function modeling.

### 2.3. Image Interpretation

Images were interpreted by board-certified nuclear medicine physicians on Oasis (Segami, Columbia, MD, USA) or AW Workstation (GE Healthcare, Chicago, IL, USA). Physicians with access to clinical data completed a qualitative interpretation case report form, recording the number of positive lesions (0, 1, 2, 3, 4, 5, 6–10, or >10) and their anatomic site (local relapse, regional nodes, distant nodes, bone, lung, or other), and listed scans as positive or negative. PET/CT images, reconstructed without the time-of-flight option, were analyzed on an MIM workstation (MIM Encore™ version 6.9.4, MIM Software Inc., Cleveland, OH, USA). Quantitative data, comprising the mean, peak, and maximum standardized uptake values adjusted for the lean body mass (SULmean, SULpeak, and SULmax) and metabolic tumor volume (MTV), together with total lesion glycolysis (TLG), of the five most active lesions of each scan were recorded using three-dimensional PET edge/brush tool running on MIM. For normal organs, regions of interest (ROIs) were drawn at predetermined reference sites including the parotid gland (1.5 cm spherical ROI centered in the right parotid gland), cardiac blood pool (2 cm spherical ROI centered in the left ventricle), and liver (3 cm spherical ROI centered in the right liver lobe).

### 2.4. Statistical Analysis

Categorical variables are presented with absolute and relative frequencies. Descriptive values are expressed as the mean (±SD) or median [range] if data were not normally distributed according to the Kolmogorov–Smirnov test. PSA-stratified detection rates were calculated as the rate of [^18^F]DCFPyL-positive results for five pooled subgroups (>0.4 to 0.5 ng/mL, 0.5 to <1.0 ng/mL, 1.0 to <2.0 ng/mL, 2.0 to <10.0 ng/mL, and ≥10.0 ng/mL). PSA-stratified detection rates were calculated with exact 95% confidence intervals. The Mann–Whitney U test and the independent t-test were used for comparison of the PSA doubling-times (PSA-DTs) and the radiotracer uptake variables between the two groups of patients with and without ADT at the time of PET/CT. Detection rates, T stages, and the Gleason scores in patients with and without ADT were evaluated using Fisher’s exact test. All tests were two-sided. Statistical significance was established for *p*-values of less than 0.05. Statistical analyses were conducted using the IBM SPSS Statistics 25.0 (IBM corporation. Armonk, NY, USA) and R (version 3.6.0; The R Foundation for Statistical Computing, General Public License).

## 3. Results

Baseline characteristics are summarized in Table 1. A total of 540 individuals were included in the analysis. Among them, 95 subjects (mean age 72.5 ± 7.7 years) were receiving ADT at the time of PET/CT, defined as the “ADT” group, and 445 patients (mean age 70.9 ± 18.5 years), who were not under ADT at the time of imaging and were classified as the “no ADT” group. ADT was present in 9 patients with PSA levels of 0.4 to 0.5 ng/mL, 14 patients with PSA levels of 0.5 to <1 ng/mL, 15 patients with PSA levels of 1 to <2 ng/mL, 26 patients with PSA levels of 2 to <10 ng/mL, and 31 patients with PSA ≥ 10 ng/mL. The median duration of ADT administration was 4 months [0.5–67 months]. In the “no ADT” group, 43 patients were enrolled with PSA between 0.4 and 0.5 ng/mL, 87 with PSA between 0.5 and <1 ng/mL, 57 with PSA between 1 and <2 ng/mL, 202 with PSA between 2 and <10 ng/mL, and 56 with PSA ≥ 10 ng/mL. Prior treatments included radical prostatectomy (44.2% of cases in the ADT group, 71.5% in the no ADT group), radiotherapy (45.3% in the ADT group, 63.6% in the no ADT group), or ADT (100% in the ADT group, 41.1% in the no ADT group), with some participants having received more than one type of therapy. The median PSA at the time of PSMA PET scanning was 3.4 ng/mL [0.44–67.5 ng/mL] and 2.71 ng/mL [0.41–71.1 ng/mL] in the ADT and “no ADT” groups, respectively. In order to exclude the treatment effects, the PSA-DT was calculated before the initiation of ADT in the ADT subgroup (median of 5 months [0.9–49.5 months]) and was significantly shorter than the PSA-DT in the “no ADT” subgroup at the time of imaging (median of 6.3 months [0–171.1 months], *p*-value = 0.038). In addition, the frequency of the prostate cancer of more aggressive behavior was significantly higher in terms of the T stage (T3 + T4) and the Gleason score (≥8) in the ADT subgroup compared to the “no ADT” subgroup (*p*-values of 0.002 and 0.004, respectively).

The detection rate of [^18^F]DCFPyL PET/CT was higher in patients receiving ADT according to Fisher’s exact test (*p*-value = 0.007). Lesions were detected in 91.6% of patients under ADT and 80.4% of patients without ADT. Evaluation of [^18^F]DCFPyL PET/CT images was feasible in all patients. In total, the certainty level of diagnosis was 2% low, 10.4% moderate, and 87.6% high in the ADT, and 5.2% low, 12.3% moderate, and 82.5% high in the no ADT group, demonstrating a good confidence of readers in their findings.

Of the 87 positive scans in the ADT group, PSMA PET/CT revealed local recurrences in 48.4% of patients, regional lymph node metastases in 46.3% of patients, bone metastases in 42.1% of patients, distant lymph node metastases in 34.7% of patients, pulmonary metastases in 4.2% of patients, and metastases in other sites in 1.0% of patients. In the group of patients without ADT (n = 445), active disease was most often identified in regional nodes (47.2%) followed by prostate bed/seminal vesicles (31.2%), distant nodes (26.7%), bone (22.5%), lung (4.0%), and other sites (1.0%) (Supplementary Table S1). A number of participants in both groups had disease in more than one site.

**Figure 1:**
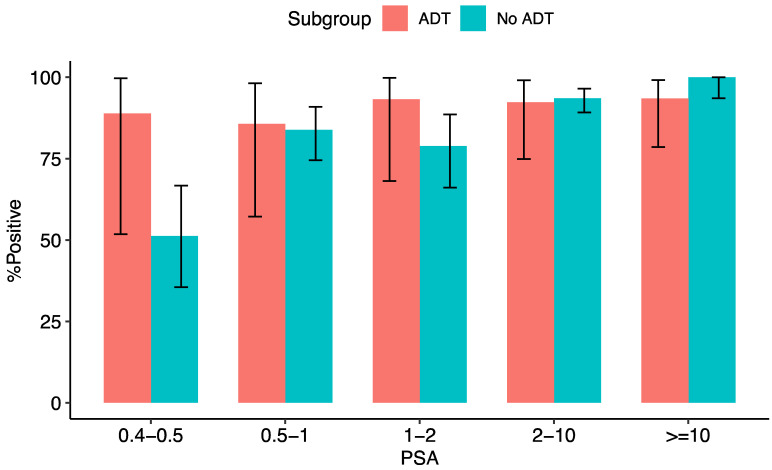
The proportion of positive scans in different PSA subgroups in patients with/without ongoing ADT at the time of PET/CT. Error bars represent 95% confidence interval.

**Figure 2:**
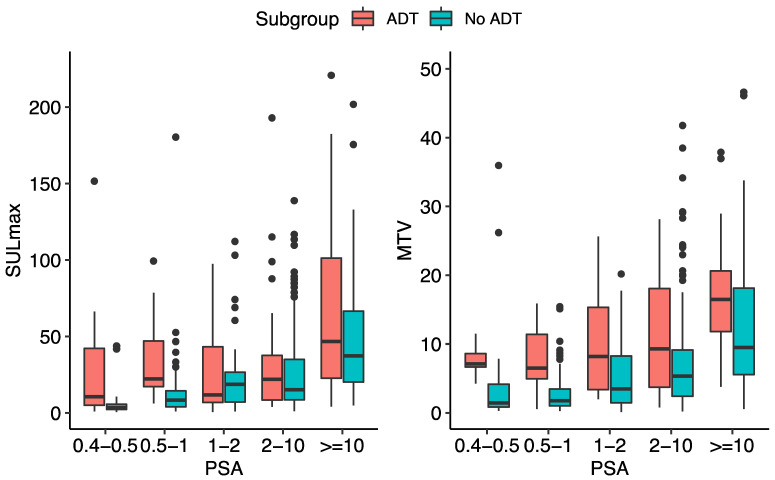
The sum of top five lesions’ SULmax and MTV in different PSA subgroups in patients with/without ongoing ADT at the time of PET/CT.

The relative PSA-stratified performance of [^18^F]DCFPyL PET/CT along with the uptake characteristics of the PSMA-positive lesions are shown in Table 2 and in Figure 1 and Figure 2. In the ADT subgroup, detection rates of [^18^F]DCFPyL PET/CT were 88.9% for PSA values of 0.4 to 0.5 ng/mL, 85.7% for PSA values of 0.5 to <1 ng/mL, 93.3% for PSA values of 1 to <2 ng/mL, 92.3% for PSA values of 2 to <10 ng/mL, and 93.5% for a PSA value of ≥10.0 ng/mL. In patients not under ADT, detection rates were 51.2%, 83.9%, 78.9%, 93.6%, and 100% for PSA levels of 0.4 to 0.5, 0.5 to <1, 1 to <2, 2 to <10, and ≥10 ng/mL, respectively. The difference in detection rates between patients with and without ADT did not reach statistical significance in the aforementioned PSA subgroups.

No significant difference was observed for radiotracer uptake in the left ventricular blood pool, liver, and parotid glands between the two groups with and without ADT (Table 3). When considering the sum of quantitative uptake characteristics of up to five lesions for each patient with PSMA-positive findings (the maximum recorded on the quantitative assessment), except for the sum of SULmax in the PSA range of 1.0 to <2.0 ng/mL, the intensity and volume of [^18^F]DCFPyL accumulation were shown to be higher in patients with ADT compared to the patients without. However, statistical significance was only attained for the sum of SULmax, sum of SULmean, and sum of SULpeak in PSA range of 0.5 to <1.0 ng/mL (*p*-values of 0.0004, 0.0001, and 0.0003, respectively); sum of TLG in PSA range of 0.4 to 0.5, 0.5 to <1.0, 2.0 to <10.0, and ≥10.0 ng/mL (*p*-values of 0.004, 0.0003, 0.02, and 0.009, respectively); and sum of MTV in PSA range of 0.4 to 0.5, 0.5 to <1.0, 1.0 to <2.0, 2.0 to <10.0, and ≥10.0 ng/mL (*p*-values of 0.009, 0.0005, 0.01, 0.03, and 0.01, respectively) (Table 2).

## 4. Discussion

This study aimed to determine the influence of ongoing ADT at the time of imaging on tumor visibility and the sensitivity of [^18^F]DCFPyL PET/CT for the detection of PCa relapse in the context of biochemical recurrence. Since studies demonstrating an increase in PSMA expression as a result of ADT administration, primarily published more than two decades ago [8], a few small clinical studies have been carried out on this issue. However, due to conflicting results of the preceding studies, there is still an ongoing discussion on how ADT affects in vivo PSMA expression and consequently clinical detection rates. This study examined a large cohort of participants that were enrolled in an investigator-initiated [^18^F]DCFPyL PET/CT imaging study.

Currently, internationally accepted guidelines miss clear-cut orientations on the issue of ADT effects on PSMA expression. It has already been suggested by in vitro studies that ADT upregulates the expression of PSMA [8,12,13]. Previous preclinical studies have shown that ADT upregulates the expression of PSMA in both androgen-sensitive and androgen-resistant human prostate adenocarcinoma cells (LNCaP) [6,14,15]. Conversely, other studies showed that the administration of ADT induced a decrease in the androgen receptor and PSMA levels in androgen-sensitive LNCaP cells [16]. An animal xenograft study also reported increased PSMA expression after ADT, while administration of enzalutamide and castration decreased tumor size [17]. The inconsistencies in the published data may partly originate from the paradoxical effects of ADT on tumor size and PSMA expression, reducing tumor mass as a result of prostate cancer cell death while increasing PSMA expression per cell at the same time. Given the fact that mechanisms involved in the upregulation of PSMA expression might in turn result in an increased PSMA PET sensitivity (and vice versa), a deepening of our knowledge of this topic would be of huge relevance.

The currently available clinical data in this regard are very heterogeneous in terms of the number and clinical status of patients enrolled, the type of ADT, and the duration of ADT administration before PSMA PET. As a result, discordant data are noted in the literature. In the first clinical study in this context, Hope et al. reported an increase in PSMA expression in prostate cancer metastases along with an increase in the number of lesions visualized using [^68^Ga]PSMA-11 PET/magnetic resonance imaging (MRI)following inhibition of the androgen receptor, albeit based on only one patient with castration-sensitive metastatic PCa [7]. In another study, Afshar-Oromieh et al. demonstrated that patients under ADT at the time of [^68^Ga]PSMA-11 PET/CT more frequently presented with a positive scan than patients not under ADT [18]. Derlin et al. assessed 34 patients with biochemical relapse of PCa under ADT and 65 patients not under ADT, and demonstrated a higher detection rate in the first group (73.5% vs. 41.5%, *p* = 0.003). By contrast, [^68^Ga]Tris(hydroxypyridinone)(THP)-PSMA uptake was comparable in patients with and without ADT (SUVmax, 8.3 ±7.4 vs. 10.7 ± 12.5, *p* = 0.18). Nevertheless, PSA values were significantly higher in patients under ADT and a PSA-stratified comparison of patients with and without ADT was not available [19]. Conversely, Eiber et al. reported no significant association between ADT administration within the last 6 months prior to the scan and the probability of a positive [^68^Ga]PSMA-11 PET/CT scan [20]. Indeed, all these observations need to be interpreted with caution as the associations observed could have been a result of either a possible biological impact of ADT (alterations in PSMA expression) or the fact that ADT might have been more frequently administered in patients with more advanced disease.

An investigation of castration-sensitive PCa patients (n = 10), conducted by Afshar-Oromieh et al., indicated that continuous long-term ADT (defined by a mean duration of 7 months) significantly reduces the visibility of tumoral lesions on [^68^Ga]PSMA-11 PET/CT [21]; however, irrespective of complete or partial PSA response, PSMA uptake was increased in 13% of lesions. The authors hypothesized that the presence of early castration-resistant cell clones might be the reason for an increased PSMA uptake in those lesions during systemic therapy. On the other hand, another PSMA PET imaging study of 37 patients, including 14 cases of metastatic castration-sensitive and 23 cases of castration-resistant prostate cancer, carried out by Mena et al., showed a lower level of PSMA uptake for early castration-sensitive patients on short-term ADT compared to those with advanced disease and long-term ADT [22]. Emmett et al. also described a 30% decrease in SUVmax after nine days of treatment with luteinizing hormone-releasing hormone (either with or without bicalutamide) in eight patients with metastatic castration-sensitive PCa along with a 45% increase in SUVmax after nine days of treatment with either enzalutamide or abiraterone in seven patients with castration-resistant PCa. The authors, therefore, speculated that ADT might exert anti-proliferative effects in the hormone-naïve stages of the disease [10]. Despite the lack of consistency in the literature, it has been suggested that imaging with PSMA-targeting PET tracers may be affected by ADT-induced early but temporarily increased PSMA expression. However, prolonged ADT may yield inconclusive imaging results due to the volumetric reduction in tumor lesions [23].

Herein, we assessed the PSA-stratified detection rates and radiotracer uptake values so as to exclude the impact of PSA level on the [^18^F]DCFPyL PET/CT performance. Of note, the overall detection rate was higher in patients with ongoing ADT at the time of PET/CT (91.6% vs. 80.4%, *p* = 0.007) in this study. However, the PSA-stratified differences in detection rates between these two groups of patients were not of statistical significance. Given the shorter PSA-DT, the higher proportions of the Gleason scores of more than 8 and disease of higher T stage (T3 + T4) in patients with ADT, and the fact that [^18^F]DCFPyL uptake was higher in ADT-receiving cases compared to those without, the higher uptake and detection rates in this study could be explained by several factors: (1) the biological effects of ADT on PSMA expression; (2) a higher tumor burden in patients under ADT at the time of PET/CT; and (3) a more aggressive clinical disease which led referring physicians to initiate ADT prior to referring participants for the PSMA PET/CT imaging trial, with failure to completely suppress PSA to undetectable levels.

No significant difference was observed for radiotracer uptake in normal organs between the two groups with and without ADT. The only other study evaluating the alterations in PSMA uptake in physiologically avid organs was carried out by Ettala et al. [24], which enrolled nine patients with treatment-naïve PCa and demonstrated an increased PSMA uptake in normal salivary glands post-ADT. They also reported a heterogeneously increased PSMA uptake in tumoral lesions post-ADT [24].

As a limitation to this study, not all patients with a positive PSMA PET scan had a histopathological reference standard to confirm that the positive findings were metastases or local recurrences. The patient-based specificity could not be calculated as a result of the absence of a histopathological reference standard and true negative patients. The wide range of ADT duration was the other constraint.

In this study, when PSA levels were above 0.4 ng/mL, patients with ongoing ADT at [^18^F]DCFPyL PET/CT more frequently presented with a positive scan than those without. Furthermore, [^18^F]DCFPyL uptake was higher in malignant lesions in patients under ADT. This may reflect a more aggressive disease phenotype in these patients, which led to earlier initiation of ADT. Our study shows that [^18^F]DCFPyL PET/CT can localize recurrence in patients with biochemically recurrent PCa under ADT even at low PSA values. The important clinical implication of our findings is that [^18^F]DCFPyL PET/CT can be performed in patients with ongoing ADT who are progressing by PSA, ideally with PSA levels above the threshold used in this study (>0.4 ng/mL), potentially allowing the localization of the progressing/resistant/prominent lesions.

## Figures and Tables

**Table 1:** Patient characteristics and treatment history.

Characteristic	ADT (n = 95)	No ADT (n = 445)
Age (years)	72.5 ± 7.7	70.9 ± 18.5
Weight (kg)	90.7 ± 20.5	87.8 ± 17.0
Height (cm)	174.4 ± 7.4	174.7 ± 7.2
Injected activity (MBq)	373.7 ± 58.2	363.9 ± 56.7
PSA at PET/CT (ng/mL), median (range)	3.4 (0.44–67.5)	2.71 (0.41–71.1)
PSA-DT * (mo), median (range)	5.0 (0.9–49.5)	6.3 (0–171.1)
Initial Gleason score		
6	7 (7.4%)	40 (9.0%)
7 (3 + 4)	14 (14.7%)	111 (24.9%)
7 (4 + 3)	29 (30.5%)	155 (34.8%)
8	9 (9.5%)	50 (11.2%)
9	36 (37.9%)	89 (20.0%)
Primary tumor classification		
p/cT1	6 (6.3%)	31 (7.0%)
p/cT2	28 (29.5%)	206 (46.3%)
p/cT3	54 (56.8%)	207 (46.5%)
p/cT4	7 (7.4%)	1 (0.2%)
Primary nodal status		
p/cN0	54 (56.8%)	304 (68.3%)
p/cN1	22 (23.2%)	43 (9.7%)
p/cNx	19 (20.0%)	98 (22.0%)
Prior treatment ^†^		
RP	42 (44.2%)	318 (71.5%)
RT	43 (45.3%)	283 (63.6%)
ADT	95 (100.0%)	183 (41.1%)
Duration of ADT (mo), median (range)	4.0 (0.5–67.0)	-

^†^ Categories are not mutually exclusive. * PSA-DT was calculated before the initiation of ADT and at the time of PET/CT in the ADT and no ADT subgroups, respectively (n = 42 in the ADT subgroup, n = 389 in the “no ADT” subgroup). PSA, Prostate-specific antigen; PSA-DT, PSA doubling-time; RP, radical prostatectomy; RT, radiation therapy; ADT, androgen deprivation therapy; PET/CT, positron emission tomography/computed tomography.

**Table 2:** [^18^F]DCFPyL PET/CT detection rates and the sum of the five most active lesions’ quantitative parameters in different PSA subgroups in patients with/without ongoing ADT at the time of PET/CT.

Parameter	Subgroup	PSA: 0.4–0.5	PSA: ≥0.5–1	PSA: ≥1–2	PSA: ≥2–10	PSA: ≥10
Detection rate (%)	ADT	88.9%	85.7%	93.3%	92.3%	93.5%
	No ADT	51.2%	83.9%	78.9%	93.6%	100.0%
	*p* value ^†^	0.06	1.0	0.27	0.68	0.12
Median [range] SULmax	ADT	10.6 [1.0, 151.5]	22.3 [6.2, 99.3]	15.8 [0.6, 308.6]	22.0 [4.1, 192.9]	48.8 [4.1, 327.9]
	No ADT	6.3 [2.1, 215.8]	8.4 [1.0, 180.4]	18.7 [1, 112.1]	15.5 [1.1, 406.2]	37.4 [4.9, 201.8]
	*p* value ^‡^	0.43	0.0004	0.89	0.48	0.09
Median [range] SULmean	ADT	4.0 [0.6, 23.7]	11.2 [4.2, 33.0]	12.3 [1.3, 126.7]	10.7 [2.1, 62.8]	22.2 [2.4, 138.6]
	No ADT	3.6 [1.4, 81.0]	4.64 [0.7, 48.4]	8.8 [0.7, 50.4]	8.4 [0.9, 174.3]	19.9 [2.2, 83.9]
	*p* value ^‡^	0.87	0.0001	0.27	0.56	0.34
Median [range] SULpeak	ADT	6.9 [0.8, 72.5]	15.6 [3.3, 63.7]	10.0 [0.4, 192.0]	13.2 [2.3, 95.4]	30.8 [3.4, 212.8]
	No ADT	4.0 [1.5, 134.0]	5.0 [0.7, 81.8]	9.8 [0.7, 0.59]	10.7 [0.8, 302.9]	24.2 [3.6, 121.2]
	*p* value ^‡^	0.25	0.0003	0.54	0.40	0.13
Median [range] TLG	ADT	14.48 [4.2, 282.4]	25.3 [2.3, 171.8]	30.5 [0.1, 723.3]	57.8 [2.7, 716.9]	129.7 [14.7, 1540.4]
	No ADT	3.4 [0.6, 435.7]	4.3 [0.4, 147.1]	13.4 [0.3, 281.2]	20.7 [0.2, 2821.8]	64.2 [3.3, 2228.4]
	*p* value ^‡^	0.004	0.0003	0.17	0.023	0.009
Median [range] MTV	ADT	7.1 [4.2, 11.5]	6.5 [0.5, 15.9]	9.4 [2.0, 60.3]	9.3 [0.8, 92.0]	16.8 [3.8, 75.1]
	No ADT	1.4 [0.3, 35.9]	1.8 [0.2, 15.4]	3.5 [0.1, 20.2]	5.5 [0.2, 81.8]	9.8 [0.5, 134.9]
	*p* value ^‡^	0.009	0.0005	0.015	0.035	0.014

^†^ *p* values from Fisher’s exact test. ^‡^ *p* values from Mann–Whitney U test.

**Table 3:** Uptake characteristics of the normal organs in patients with/without ongoing ADT at the time of PET/CT.

Variable	ADT (n = 95)	No ADT (n = 445)	*p* Value ^†^
Cardiac blood pool SULmean	0.8 ± 0.1	0.8 ± 0.2	0.685
Liver SULmean	4.9 ± 1.1	4.8 ± 0.9	0.488
Parotid gland SULmean	10.4 ± 3.0	11.2 ± 3.5	0.051

^†^ *p* values from the independent *T* test.

## Data Availability

The data presented in this study are available on request from the corresponding author. The data are not publicly available due to ethical considerations.

## Supplementary Materials

The following supporting information can be downloaded at: https://www.mdpi.com/article/10.3390/diagnostics12092212/s1, Table S1: Qualitative assessment of scans based on PSA levels.
