# Supplementary material for: PSA-Stratified Performance of [18F]DCFPyL PET/CT in Biochemically Recurrent Prostate Cancer Patients under Androgen Deprivation Therapy
Source: Diagnostics (Basel). 2022 Sep 13;12(9):2212. doi: 10.3390/diagnostics12092212 (PMC9498260; doi:10.3390/diagnostics12092212)
Supplement: Supplementary file 1 [file diagnostics-12-02212-s001.zip › diagnostics-1878779-supplementary.pdf]

Supplemental Table S1. Qualitative assessment of scans based on PSA levels.

Parameter	Subgroup	PSA: 0.4-0.5	PSA: ≥ 0.5-1	PSA: ≥ 1-2	PSA: ≥ 2-10	PSA: ≥ 10
**Patients**	ADT (N=95)	9 (9.5%)	14 (14.7%)	15 (15.8%)	26 (27.4%)	31 (32.6%)
	No ADT (N=445)	43 (9.7%)	87 (19.5%)	57 (12.8%)	202 (45.4%)	56 (12.6%)
**Number of lesions**						
**0**	ADT	1 (1.0%)	2 (2.1%)	1 (1.0%)	2 (2.1%)	2 (2.1%)
	No ADT	21 (4.7%)	14 (3.1%)	12 (2.7%)	13 (2.9%)	0 (0%)
**1**	ADT	2 (2.1%)	1 (1.0%)	5 (5.3%)	10 (10.5%)	4 (4.2%)
	No ADT	11 (2.5%)	39 (8.8%)	19 (4.3%)	72 (16.2%)	14 (3.1%)
**2**	ADT	1 (1.0%)	2 (2.1%)	1 (1.0%)	3 (3.2%)	5 (5.3%)
	No ADT	4 (0.9%)	13 (2.9%)	6 (1.3%)	28 (6.3%)	8 (1.8%)
**3**	ADT	2 (2.1%)	2 (2.1%)	2 (2.1%)	4 (4.2%)	1 (1.0%)
	No ADT	3 (0.7%)	9 (2.0%)	5 (1.1%)	21 (4.7%)	2 (0.4%)
**4**	ADT	1 (1.0%)	1 (1.0%)	1 (1.0%)	1 (1.0%)	0 (0%)
	No ADT	0 (0%)	3 (0.7%)	2 (0.4%)	10 (2.2%)	0 (0%)
**5**	ADT	0 (0%)	0 (0%)	0 (0%)	0 (0%)	1 (1.0%)
	No ADT	0 (0%)	3 (0.7%)	4 (0.9%)	10 (2.2%)	5 (1.1%)
**6-10**	ADT	2 (2.1%)	4 (4.2%)	4 (4.2%)	2 (2.1%)	6 (6.3%)
	No ADT	2 (0.4%)	3 (0.7%)	6 (1.3%)	24 (5.4%)	3 (0.7%)
**> 10**	ADT	0 (0%)	2 (2.1%)	1 (1.0%)	4 (4.2%)	12 (12.6%)
	No ADT	1 (0.2%)	3 (0.7%)	3 (0.7%)	24 (5.4%)	24 (5.4%)
**Site of lesions**†						
**Local relapse**	ADT	3 (3.2%)	3 (3.2%)	5 (5.3%)	16 (16.8%)	19 (20.0%)
	No ADT	9 (2.0%)	18 (4.0%)	5 (1.1%)	78 (17.5%)	29 (6.5%)
**Regional nodes**	ADT	2 (2.1%)	8 (8.4%)	4 (4.2%)	11 (11.6%)	19 (20.0%)
	No ADT	10 (2.2%)	44 (9.9%)	36 (8.1%)	91 (20.4%)	29 (6.5%)
**Distant nodes**	ADT	2 (2.1%)	5 (5.3%)	4 (4.2%)	5 (5.3%)	17 (17.9%)
	No ADT	4 (0.9%)	13 (2.9%)	10 (2.2%)	59 (13.3%)	33 (7.4%)
**Bone**	ADT	5 (5.3%)	3 (3.2%)	9 (9.5%)	8 (8.4%)	15 (15.8%)
	No ADT	4 (0.9%)	17 (3.8%)	10 (2.2%)	47 (10.6%)	22 (4.9%)
**Lung**	ADT	0 (0%)	1 (1.0%)	0 (0%)	2 (2.1%)	1 (1.0%)
	No ADT	2 (0.4%)	2 (0.4%)	0 (0%)	10 (2.2%)	4 (0.9%)
**Other**	ADT	0 (0%)	0 (0%)	1 (1.0%)	0 (0%)	0 (0%)
	No ADT	1 (0.2%)	1 (0.2%)	0 (0%)	2 (0.4%)	1 (0.2%)
**Diagnosis**						
**Negative**	ADT	1 (1.0%)	2 (2.1%)	1 (1.0%)	2 (2.1%)	2 (2.1%)
	No ADT	21 (4.7%)	14 (3.1%)	12 (2.7%)	13 (2.9%)	0 (0%)
**Positive**	ADT	8 (8.4%)	12 (12.6%)	14 (14.7%)	24 (25.3%)	29 (30.5%)
	No ADT	22 (4.9%)	73 (16.4%)	45 (10.1%)	189 (42.5%)	56 (12.6%)
**Certainty of diagnosis**						
**Low**	ADT	1 (1.0%)	0 (0%)	0 (0%)	0 (0%)	1 (1.0%)
	No ADT	1 (0.2%)	11 (2.5%)	3 (0.7%)	8 (1.8%)	0 (0%)
**Moderate**	ADT	1 (1.0%)	1 (1.0%)	3 (3.2%)	4 (4.2%)	1 (1.0%)
	No ADT	9 (2.0%)	14 (3.1%)	4 (0.9%)	27 (6.1%)	1 (0.2%)
**High**	ADT	7 (7.4%)	13 (13.7%)	12 (12.6%)	22 (23.2%)	29 (30.5%)
	No ADT	33 (7.4%)	62 (13.9%)	50 (11.2%)	167 (37.5%)	55 (12.4%)

† Categories are not mutually exclusive.
